# Supplementary material for: A bend, flip and trap mechanism for transposon integration
Source: eLife. 2016 May 25;5:e15537. doi: 10.7554/eLife.15537 (PMC5481204; doi:10.7554/eLife.15537)
Supplement: Figure 3—source data 1. — DOI: http://dx.doi.org/10.7554/eLife.15537.011 [file elife-15537-fig3-data1.docx]

**Figure 3–source data 1. Fluorescence decay parameters for 2AP-containing duplexes, TP13 and TP1, in the absence and presence of Mos1 transposase.** The fluorescence lifetimes, τ_i_, and their corresponding A-factors, A_i_, are given, where the latter indicate the fractional population of each lifetime component. The quality of the fits was judged on the basis of the reduced chi-squared statistic and the randomness of residuals. Results from three repeat measurements were in good agreement, and the uncertainties in reported values of lifetimes and A-factors were ≤10%. Related to Figure 3.

| **Sample** | **τ_1_/ns** | **τ_2_/ns** | **τ_3_/ns** | **τ_4_/ns** | **A_1_** | **A_2_** | **A_3_** | **A_4_** |
| --- | --- | --- | --- | --- | --- | --- | --- | --- |
| **TP13** | 0.03 | 0.39 | 2.5 | 8.7 | 0.90 | 0.04 | 0.05 | 0.01 |
| **TP13 + Mos1** | 0.03 | 0.36 | 2.5 | 8.5 | 0.90 | 0.04 | 0.05 | 0.01 |
| **TP1** | 0.05 | 0.46 | 2.7 | 7.5 | 0.76 | 0.10 | 0.08 | 0.06 |
| **TP1 + Mos1** | 0.08 | 0.36 | 3.2 | 9.7 | 0.38 | 0.15 | 0.16 | 0.31 |
